# Drug Design of Cyclin-Dependent Kinase 2 Inhibitor for Melanoma from Traditional Chinese Medicine

**DOI:** 10.1155/2014/798742

**Published:** 2014-06-19

**Authors:** Hsin-Chieh Tang, Calvin Yu-Chian Chen

**Affiliations:** ^1^Department of Biomedical Informatics, Asia University, Taichung 41354, Taiwan; ^2^Department of Medicine, China Medical University, Taichung 40402, Taiwan; ^3^Research Center for Chinese Medicine & Acupuncture, China Medical University, Taichung 40402, Taiwan; ^4^Human Genetic Center, Department of Medical Research, China Medical University Hospital, Taichung 40447, Taiwan

## Abstract

One has found an important cell cycle controller. This guard can decide the cell cycle toward proliferation or quiescence. Cyclin-dependent kinase 2 (CDK2) is a unique target among the CDK family in melanoma therapy. We attempted to find out TCM compounds from TCM Database@Taiwan that have the ability to inhibit the activity of CDK2 by systems biology. We selected Tetrahydropalmatine, Reserpiline, and (+)-Corydaline as the candidates by docking and screening results for further survey. We utilized support vector machine (SVM), multiple linear regression (MLR) models and Bayesian network for validation of predicted activity. By overall analysis of docking results, predicted activity, and molecular dynamics (MD) simulation, we could conclude that Tetrahydropalmatine, Reserpiline, and (+)-Corydaline had better binding affinity than the control. All of them had the ability to inhibit the activity of CDK2 and might have the opportunity to be applied in melanoma therapy.

## 1. Introduction

One has discovered an important cell cycle controller. This gatekeeper can decide the cell cycle toward proliferation or quiescence [1]. The cell cycle means division and duplication of the cells. If the process occurs in prokaryotes, it is termed binary fission. In eukaryotes, the process can consist of interphase and mitotic (M) phase. The interphase can be further divided into G1 (gap 1) phase, S (synthesis) phase, and G2 phase [2, 3]. Normal cell cycle follows the ordinary steps, but cancer cells grow without regulation. The rate of progress in cell cycle is decided by cyclins and cyclin-dependent kinases (CDKs). Entering of each phase is controlled by specific cyclin-CDK complex. CDK is a member of serine-threonine kinase family because a cyclin binds to a CDK and starts the phosphorylation of its serine and threonine site [4, 5]. Cyclin controls the activity of CDK. In other words, CDK is like the engine in a car, and cyclin is like the gearbox. Cyclin E-CDK2 complex guides the process from G1 to S phase, while cyclin A-CDK2 complex is required to pass through the S phase [6, 7]. Related efforts let Hartwell et al., Bandara et al., and Nurse win the Nobel Prize in Physiology or Medicine 2001 [8–10].

As mention to inhibitory mechanism, the genes of kinase inhibitory protein/CDK interacting protein (kip/cip) family prevent the progression of the cell cycle. Because these proteins are produced in prevention of tumor formation, they are known as tumor suppressors. The kip/cip gene family includes the genes p21, p27, and p57. These proteins arrest cell cycle in G_1_ phase by binding to cyclin-CDK complexes and inactivating them. P21, encoded by the CDKN1A gene, is activated by p53 which plays a role in apoptosis; p27, encoded by the CDKN1B gene, is activated by transforming growth factor *β* (TGF *β*) which is a growth inhibitor; p57, encoded by the CDKN1C gene, is a negative regulator of cell proliferation [11–15]. Cancer cells are loss of cell cycle rhythm. CDK2 is encoded by CDK2 gene as a downstream product of microphthalmia-associated transcription factor (MITF) in melanocytes, too. MITF is essential for development of embryonic melanocytes and even malignant melanoma [16]. CDK2 has an important role in the occurrence and progression of melanoma among its CDK family. Inhibition of CDK2 significantly reduced growth of melanoma cells [17]. These researches have told us that CDK2 would be a unique target rather than other CDKs in melanoma therapy.

Malignant melanoma is very dangerous if it is not diagnosed and treated early. It causes high mortality rate [18]. Gold standard of primary melanoma is surgery; but combined therapy, such as chemotherapy, immunotherapy, or radiotherapy, is necessary to advanced or metastatic melanoma [19, 20]. The Raf protein/mitogen-activated protein kinase/extracellular-signal-regulated kinase (RAF/MAPK/ERK) signal pathway has thus become a molecular target for therapeutic design of advanced melanoma harboring the B-RAF gene mutation [21, 22]. However, not every melanoma lesion carries this gene mutation. In addition, resistance to RAF inhibitors has been reported recently [23–26]. Besides melanoma, CDK2 is also overexpressed in other tumors [27, 28]. One study has demonstrated a significant increase of cyclin E and CDK2 expression during tumor progression in malignant melanoma compared to benign melanocytic lesions [29]. Previous studies had demonstrated that Dinaciclib was a CDK1/2/5/9 inhibitor leading to tumor apoptosis via p53 expression [30, 31].

In this study, we aimed to determine the small molecules binding and inhibiting the function of CDK2 that would be an effective method to interfere with the aggressive biological behavior of advanced melanoma. Knowing the mechanism of various diseases provides us with the new direction to resolve them [32, 33]. Modern technology in medicine helps us be more confident in managing troublesome diseases [34]. Computational simulation has rapidly emerged in small molecular drug design [35–38]. Traditional Chinese Medicine (TCM) has mild features, and it has therapeutic effect in series of diseases [39–41]. Application of TCM database lets it become possible to find out drug-like molecules [42–44]. In this purpose, we attempted to find out candidate compounds from the largest TCM Database@Taiwan (http://tcm.cmu.edu.tw/) in the world that have the ability to inhibit the activity of CDK2 [45].

## 2. Materials and Methods

### 2.1. Docking and Candidate Screening

All small molecular compounds were downloaded from TCM Database@Taiwan (http://tcm.cmu.edu.tw/) to identify potential CDK2 inhibitor screening [45]. Cyclin-dependent kinase 2 (CDK2) protein data and structure were obtained from the Uniprot Knowledgebase (CDK2_Human, P24941) and Protein Data Bank (PDB ID: 1URW). The resolution of its crystal structure was from residue 1 to 298, and key residues of the binding sites are located at Lys33, Asp86, Asp127, Asn132, and Asp145 [46]. PONDR-FIT program in the DisProt website was employed to exclude the disordered residues of 3D structure of CDK2 [47, 48]. This experiment utilized the LigandFit program of Discovery Studio (DS) 2.5 to filter out the small molecules from TCM database that could dock with CDK2 binding sites. All the small molecules for virtual screening had passed through Lipinski's Rule of Five, absorption, distribution, metabolism, excretion, and toxicity (ADMET), to rule out potential toxic compounds in DS 2.5 [49, 50]. The locations of binding sites were originally at the ligand, imidazo(1,2-b)pyridazines or I1p (N-[3-(dimethylamino)propyl]-4-[(4-imidazo[1,2-b]pyridazin-3-yl-2-pyrimidinyl)amino]benzenesulfonamide), binding with CDK2 crystal structure. All the poses of small molecules in docking process were minimized by the force field of Chemistry at HARvard Macromolecular Mechanics (CHARMm) [51]. We also adopted the LIGPLOT program to illustrate hydrogen bond (H-bond) and hydrophobic contact between the ligand and CDK2 protein [52, 53].

### 2.2. Support Vector Machine (SVM), Multiple Linear Regression (MLR) Models, and Bayesian Network

We obtained 27 compounds and pIC50 data of CDK2 inhibitors from the study of Tripathi et al. [54]. Then we drew 2D and 3D structure of these compounds by ChemBioDraw software. Then we utilized Genetic Function Approximation (GFA) algorithm in DS 2.5 to find the appropriate molecular descriptors [41, 55]. The descriptors constructing multiple linear regression (MLR) and support vector machine (SVM) models were validated by Matlab Statistics Toolbox and libSVM. The description normalized between [−1, +1] by SVM training model. We utilized the activity of square correlation coefficient (*R* ^2^) to validate each model. The data of these compounds was adopted for predicting the control and top 3 candidate compounds. We utilized fivefold cross validation and chose the highest *R* ^2^ of SVM and MLR to perform activity prediction models.

For a well-defined Bayesian network, our algorithm was used in Matlab codes that integrated the Bayes Net Toolbox (BNT) package and the Banjo package to predict pIC50 value. The physiochemical properties relating to the binding strength were extracted as descriptors by DS 2.5.

### 2.3. Molecular Dynamics (MD) Simulation

We utilized GROningen MAchine for Chemical Simulations (GROMACS) 4.0.733 for MD simulation of the candidates and the control compound [56]. Minimization, heating, equilibration, and production were the four phases for selected protein-ligand complex simulation. We analyzed the trajectory figures of root mean square deviation (RMSD), Gyrate, mean square deviation (MSD), and solvent accessible surface area (SASA). We illustrated each ligand and its corresponding protein change for the 3 candidates and the control. Total energy, root mean square fluctuation (RMSF), RMSD matrix and clustering diagram, and secondary structure changes were adopted to compare the changes of the 3 candidates and the control during MD [57]. We calculated distance of hydrogen bond (H-bond) and its stability by torsion analysis between the ligand and essential amino acids of CDK2. Best distance of H-bond was set at 0.3 nm or 3 Å. CAVER software was adopted to analyze all possible pathways when the ligand bound with CDK2 protein [58]. The parameters were time_sparsity 1; first_frame 0; last_frame 100; probe_radius 0.9, shell_radius 4, shell_depth 5.

## 3. Results and Discussion

### 3.1. Docking and Candidate Screening

**Figure 1:**
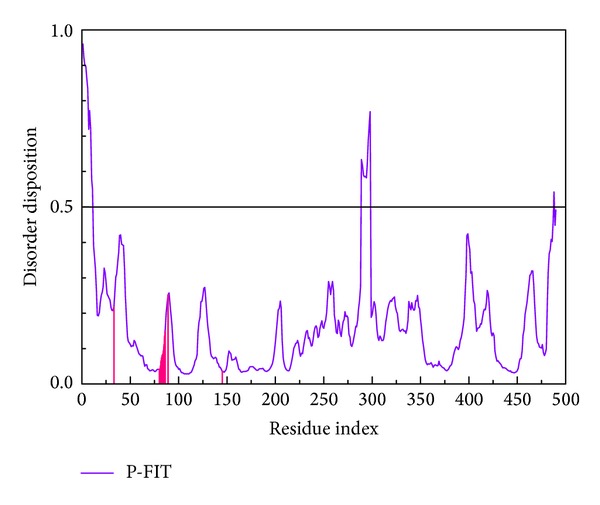
Disorder disposition of CDK2 structure. All the regions for key residues of CDK2 are in the nondisordered region (below 0.5).

**Figure 2:**
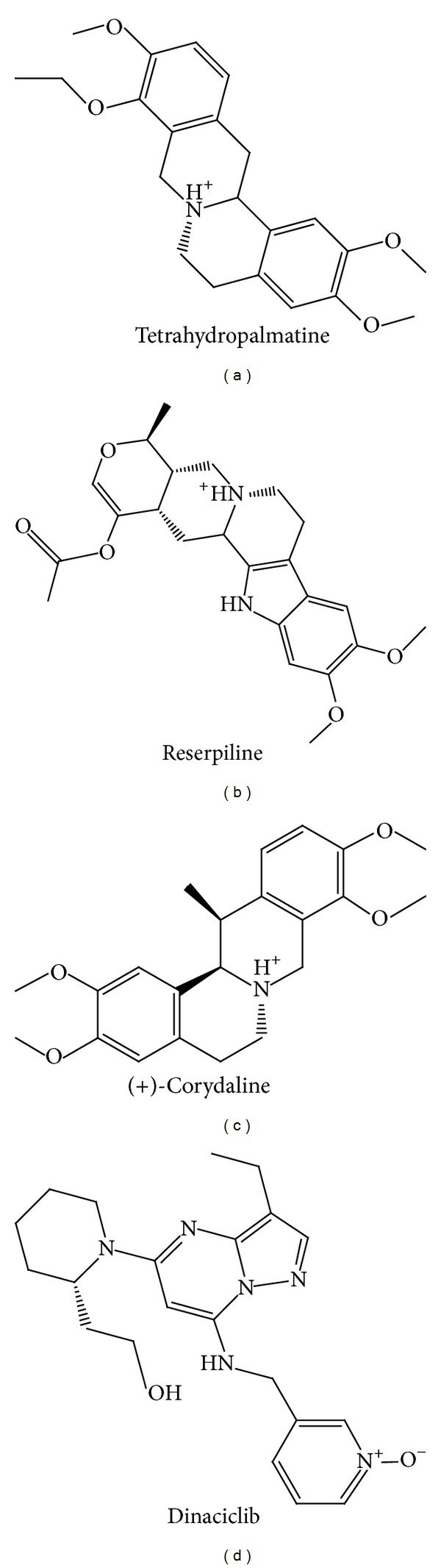
Scaffold of the top 3 TCM candidates: (a) Tetrahydropalmatine, (b) Reserpiline, (c) (+)-Corydaline, and (d) the control: Dinaciclib.

All the regions for key residues (Lys33 to Asp145) of CDK2 protein recorded in the literature did not locate at the disordered region. We could prove that the 3D structure of CDK2 (PDB ID: 1URW) was reliable (Figure 1).  Table 1 listed dock score, H-bond forming residues, H-bond quantity, SVM, MLR, and BNT of the top 10 TCM compounds ranked by dock score. We selected Tetrahydropalmatine, Reserpiline, and (+)-Corydaline as the candidates for further examination. Dinaciclib, the CDK2 inhibitor, was assigned as the control compound in this study. Tetrahydropalmatine, Reserpiline, and (+)-Corydaline were mainly extracted from* Phellodendron amurense*,* Rauwolfia serpentina*, and* Corydalis yanhusuo*, respectively. The literature had proved that the original plants of top 3 compounds had antitumor efficacy [59–62]. Therefore, we believed that the top 3 candidate compounds had the potential role in the inhibition of tumor growth. The structure of top 3 TCM compounds and control compound was shown in Figures 2(a)–2(c) and 2(d). The candidate compounds which had good affinity with binding sites according to scoring function may be associated with H-bond, charge interaction, *π* bond, van der Waals forces, and hydrophobic contact.

**Figure 3:**
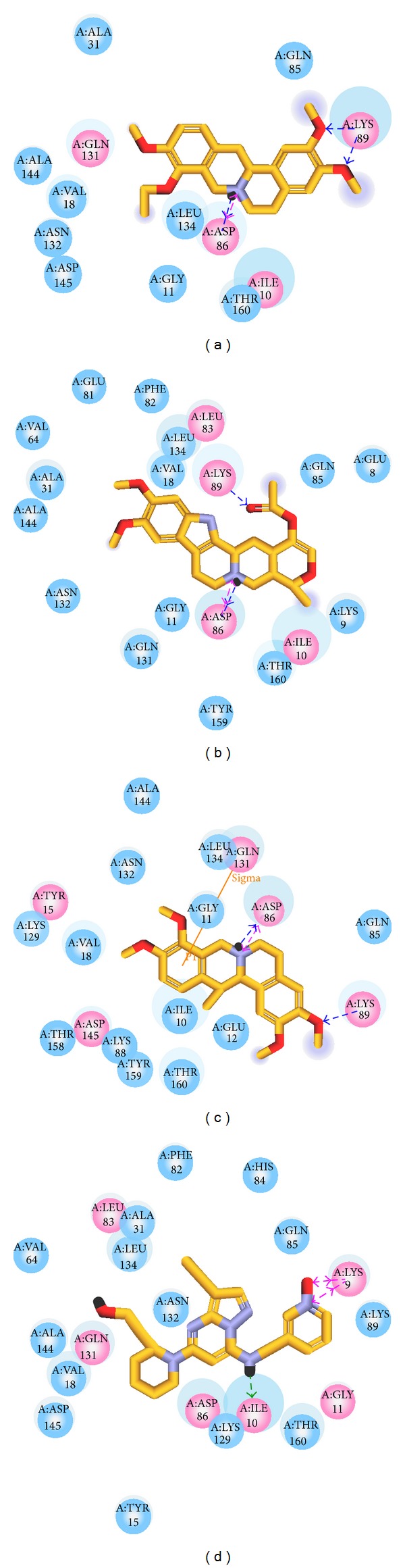
Docking poses of the ligands with CDK2 binding sites. (a) Tetrahydropalmatine, (b) Reserpiline, (c) (+)-Corydaline, and (d) Dinaciclib. Pink dashed line: charge interaction; green dashed line: H-bond with amino acids main chains; blue dashed line: H-bond with amino acids side-chains; orange line: *π* bond.

**Figure 4:**
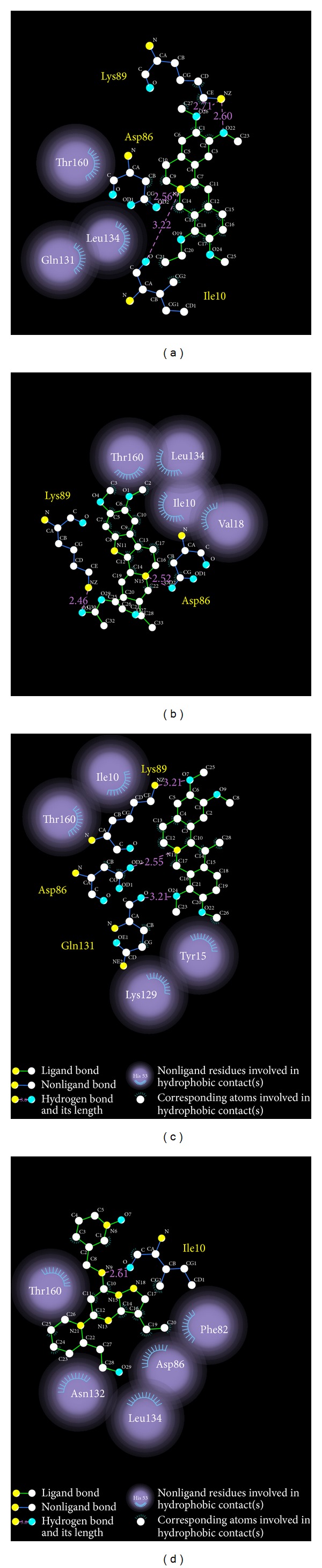
Docking poses of the ligands with CDK2 binding sites. (a) Tetrahydropalmatine, (b) Reserpiline, (c) (+)-Corydaline, and (d) Dinaciclib.

We illustrated how the top 3 and control compounds interacted with the binding sites of target protein. All the top 3 candidate compounds bound to Asp86 and Lys89 residues and formed charge interaction with Asp86. The phenomenon was consistent with the key residues described in the literature. According to this finding, Asp86 and Lys89 residues were important binding sites. (+)-Corydaline formed *π* bond with Gln131, too. The control formed H-bond with Ile10 and charge interaction with Lys9 (Figure 3).  Figure 2 showed that the candidates and the control formed hydrophobic contacts in the binding sites in addition to H-bonds. The candidate and control compounds formed hydrophobic contact with at least 3 amino acid residues, respectively. The same amino acid residue was Thr160. Tetrahydropalmatine, Reserpiline, and control compound formed hydrophobic contact with Leu134. Reserpiline and (+)-Corydaline formed hydrophobic contact with Ile10, too. Although control compound did not form H-bond with any key residue, it formed hydrophobic contact with Asp86 and Asn132 (Figure 4).

Based on the results of docking, we concluded that candidate compounds had more stable force than control compound. The hydrophobic contact of candidate compounds was less than control compound, but all of them formed hydrophobic contact with amino acid residue Thr160. The analytic result of binding sites was compatible with the trend in dock score (Table 1). We proved that Asp86 was important in the binding site again.

### 3.2. Support Vector Machine (SVM), Multiple Linear Regression (MLR) Models, and Bayesian Network

**Figure 5:**
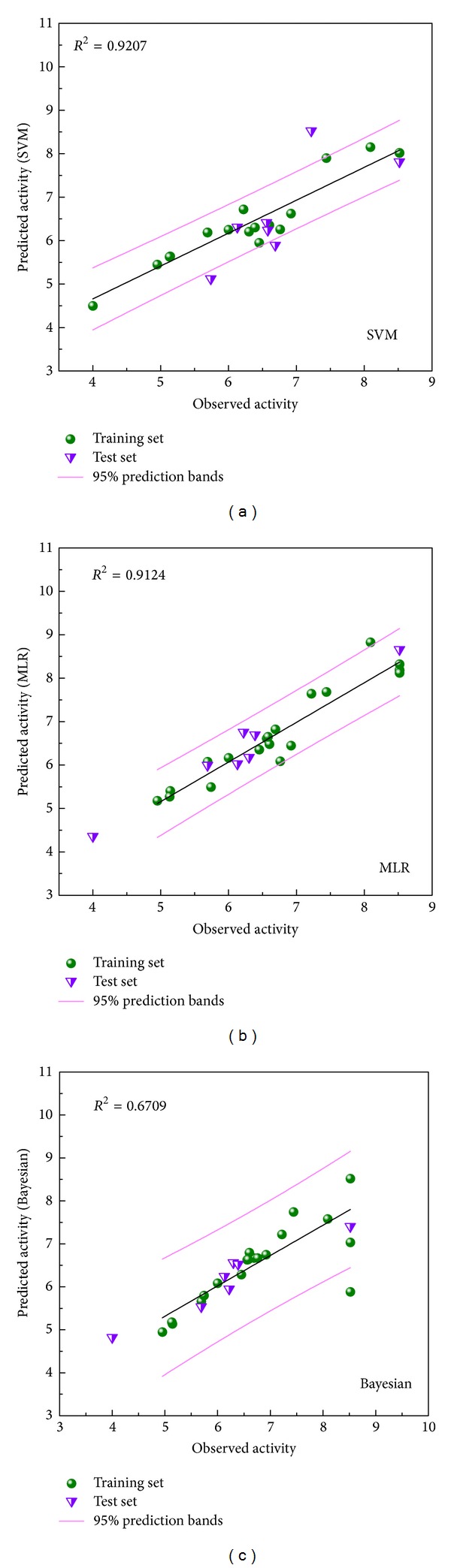
20 training sets and 7 test sets using SVM, MLR models and Bayesian network for predicted activity. *R* ^2^ of SVM = 0.9207, MLR = 0.9124, and Bayesian = 0.6709.

We selected the following 7 optimum descriptors for predicting activities: ALogP, Num_Hydrogens, Molecular_Volume, CHI_3_C, CHI_V_3_C, JY, and Jurs_RPSA. We employed these descriptors for constructing SVM, MLR models, and Bayesian network. For the 7 descriptors in this study, each set of ligand-compound discrete data allowed us to estimate complex relationships, the descriptors, and the binding strength, without hypothesis of data distribution that may bias the Bayesian network inference model. Using these descriptors, the predictive models were generated as follows: p(IC50) = −10.551 − 0.406∗ALogP − 0.776 ∗ Num_Hydrogens + 0.095 ∗ Molecular_Volume + 5.280 ∗ CHI_3_C − 2.794 ∗ CHI_V_3_C + 6.356 ∗ JY − 40.920 ∗ Jurs_RPSA. For this purpose, we discretized the data of pIC50 and these descriptors from continuous values into various categories on the basis of their distribution property. The 27 ligands of CDK2 inhibitors were randomly divided into 20 training sets and 7 test sets for validation. The *R* ^2^ for predicted biological activity of SVM, MLR, and Bayesian network were 0.9207, 0.9124, and 0.6538, respectively. These results suggested that predicted activity of any given compound was almost consistent with observed activity. SVM of Tetrahydropalmatine, Reserpiline, and (+)-Corydaline were 6.233, 7.148, and 6.217. MLR of the 3 candidates were 6.156, 6.044, and 6.283. BNT of the 3 candidates were 6.509, 6.995, and 6.499. SVM, MLR, and BNT of the control were 6.405, 3.229, and 6.899. Predicted activities of the 3 candidates were almost the same as or better than the control (Figure 5).

### 3.3. Molecular Dynamics (MD) Simulation

**Figure 6:**
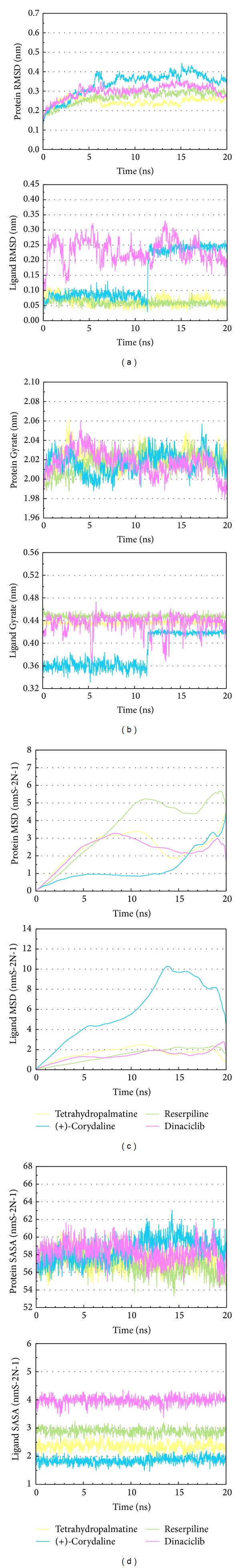
Analysis of MD trajectories generated by Gromacs. (a) RMSD, (b) Gyrate, (c) MSD, and (d) SASA.

We drew the trajectories of ligand and protein RMSD to show the deviation of each ligand and its corresponding protein during the period of MD. Interestingly, (+)-Corydaline had large deviation at 11 ns of MD, but it became stable after the large deviation. In contrast, Tetrahydropalmatine and Reserpiline were stable during the whole period of MD. However, the control was unstable during the whole period of MD. In contrast to ligand RMSD, protein RMSD of the 3 candidates and the control were relatively stable after 6 ns of MD. (+)-Corydaline corresponding protein had the largest mean RMSD value, and Tetrahydropalmatine corresponding protein had the smallest mean RMSD value. We concluded that the 3 candidates could bind with CDK2 more stably than the control (Figure 6(a)). The trajectories of ligand and protein Gyrate were drawn to show the average distance of atoms to the center of each ligand and its corresponding protein. It showed the compact degree of each ligand and its corresponding protein. Similar to ligand RMSD of (+)-Corydaline, it had large change at 11 ns of MD but became stable after the large change. In contrast, Tetrahydropalmatine and Reserpiline were stable during the whole period of MD. However, the control was unstable during the whole period of MD. In contrast to ligand Gyrate, protein Gyrate of the 3 candidates and the control was fluctuated during the whole period of MD. It was evident that all the 3 candidates and the control could induce compact change of CDK2 (Figure 6(b)). We drew the trajectories of ligand and protein MSD to show the deviation of atoms from the beginning to the end of MD. Interestingly, (+)-Corydaline had steep rise after 11 ns of MD. However, it had steep drop after 19 ns of MD and diminished the gap between the other ligands. In contrast to ligand MSD, protein MSD of Reserpiline had the largest mean MSD value. We speculated that the 3 candidates could bind with CDK2 as the control successfully despite the different patterns of MSD (Figure 6(c)). The trajectories of ligand and protein SASA were drawn to show the surface area in contact with water of each ligand and its corresponding protein. Ligand SASA of the 3 candidates and the control were stable during the whole period of MD. In contrast to ligand SASA, protein SASA of the 3 candidates and the control were fluctuated during the whole period of MD. It was evident that all the 3 candidates and the control could induce surface change of CDK2 (Figure 6(d)). According to the figures of RMSD, Gyrate, MSD, and SASA, we concluded that the 3 candidates could bind with CDK2 and induce its conformational change the same as or even more stable than the control.

**Figure 7:**
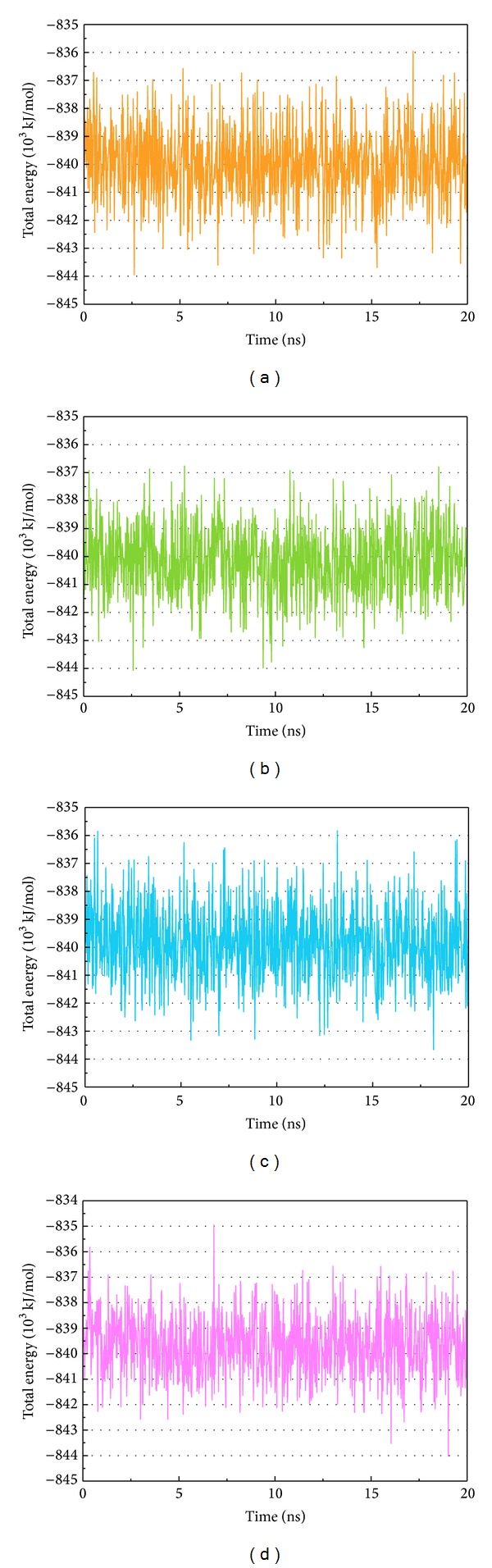
Total energy. (a) Tetrahydropalmatine, (b) Reserpiline, (c) (+)-Corydaline, and (d) Dinaciclib.

**Figure 8:**
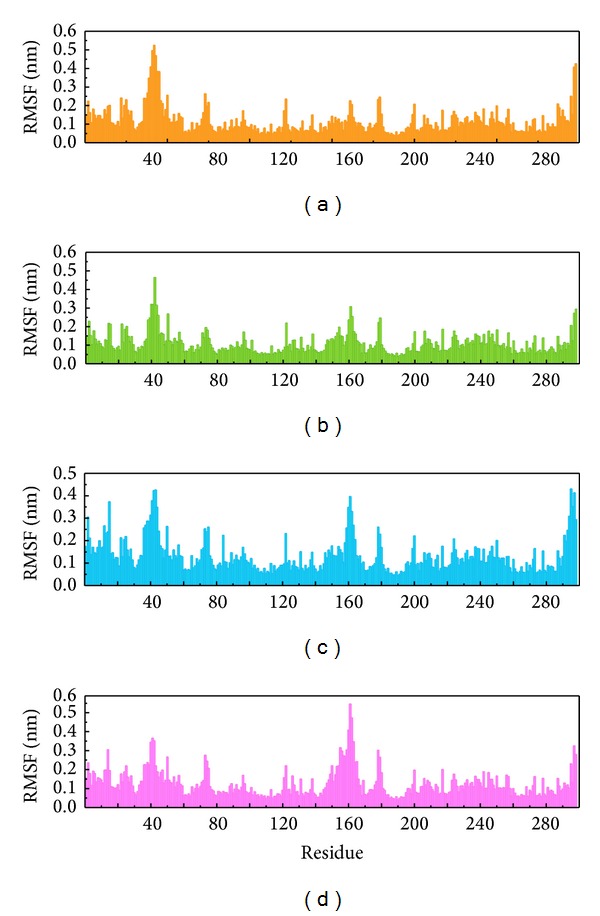
RMSF. (a) Tetrahydropalmatine, (b) Reserpiline, (c) (+)-Corydaline, and (d) Dinaciclib.

We illustrated total energy to observe the binding energy stability for the ligand and protein. The average total energy of ligand-protein complex for Tetrahydropalmatine, Reserpiline, (+)-Corydaline, and the control was −840000, −840000, −839500, and −839500 KJ/mol, respectively. The results of total energy were almost the same for the 3 candidates and the control (Figure 7). RMSF was drawn to calculate the fluctuation degree of every residue of CDK2 protein during MD. The largest fluctuation of the 3 candidates was near residue 40. However, the largest fluctuation of the control was near residue 160. Interestingly, even the line graph of RMSF was similar, but not the same for the 3 candidates (Figure 8). We concluded that all the 3 candidates and the control bound with CDK2 protein stably but caused different fluctuation in individual residues.

**Figure 9:**
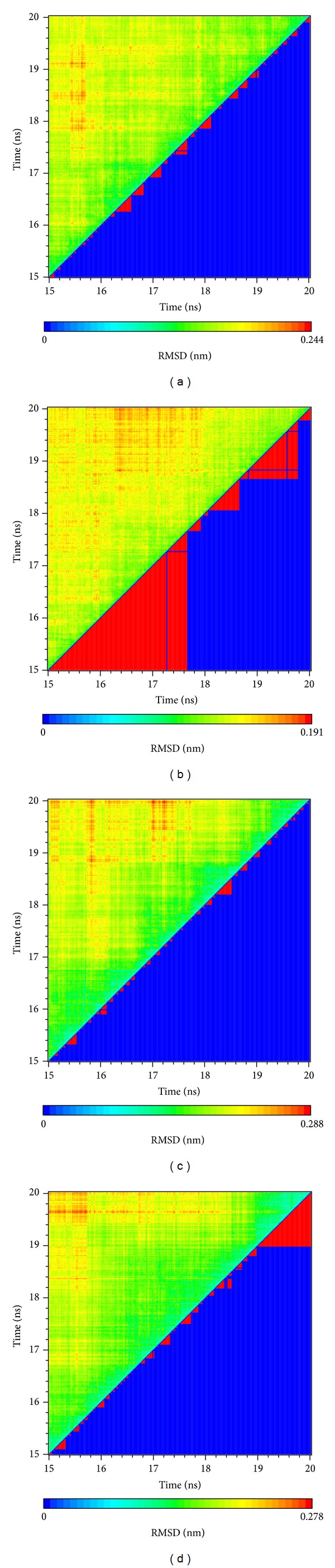
RMSD matrix and clustering diagram of MD conformations from 15 to 20 ns. (a) Tetrahydropalmatine, (b) Reserpiline, (c) (+)-Corydaline, and (d) Dinaciclib.

**Figure 10:**
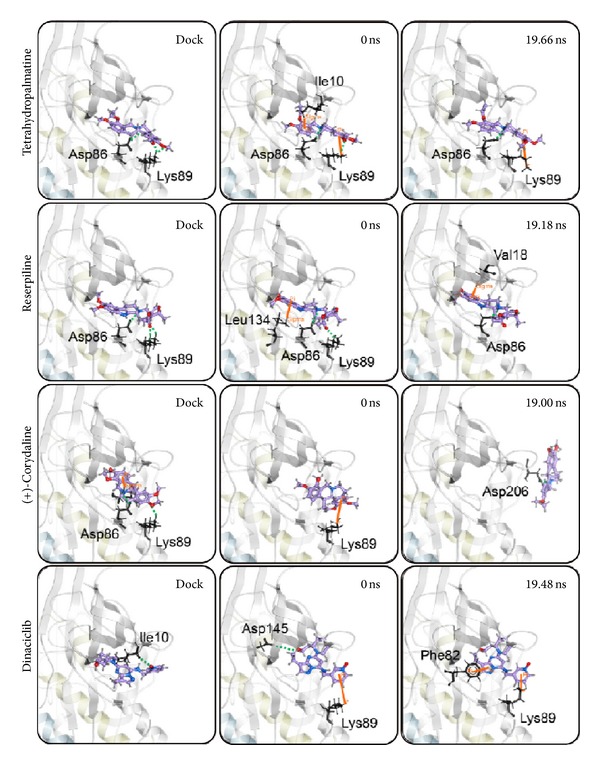
Snapshots of the ligand bound with CDK2 protein during docking and MD simulation.

We illustrated RMSD matrix and clustering diagram of MD conformations from 15 to 20 ns to find the representative structure in the period. The upper left part demonstrated RMSD values from 15 to 20 ns. The lower right part showed several stable clusters in the same period (Figure 9). According to clustering diagram after 19 ns to the end of MD, we selected 19.66, 19.18, 19.00, and 19.48 ns as the snapshot of representative structure for Tetrahydropalmatine, Reserpiline, (+)-Corydaline, and the control. The MD poses of 0 ns and snapshot after 19 ns were compared with docking poses of the ligand and CDK2 protein in Figure 10. The docking poses of the 3 candidates showed the common key residues only (Asp86 and Lys89). Tetrahydropalmatine formed additional *π* bond with Ile10 at 0 ns of MD when compared with docking poses. Reserpiline formed additional *π* bond with Leu134 at 0 ns of MD when compared with docking poses. Tetrahydropalmatine and Reserpiline still formed H-bond with Asp86 at 19.66 and 19.18 ns of MD. Reserpiline formed additional *π* bond with Val18 at 19.18 ns. (+)-Corydaline only formed *π* bond with Lys89 at 0 ns of MD when compared with docking poses and formed H-bond with Asp206 at 19 ns as a result of ligand change in direction. This finding proved the large deviation of (+)-Corydaline in the ligand RMSD at 11 ns and the steep drop in the ligand MSD of Figure 6 after 19 ns. We speculated that the direction of ligand had changed since 11 ns. After changing its direction, the ligand became stable at the end of MD. The control (Dinaciclib) was the other dramatic ligand when comparing MD poses with docking poses. The H-bond with Ile10 which originally existed in docking pose disappeared during MD. The control formed *π* bond with Lys89 and H-bond with Asp145 at 0 ns of MD in substitute. It formed *π* bond with Phe82 and Lys89 at 19.48 ns of MD (Figure 10).

**Figure 11:**
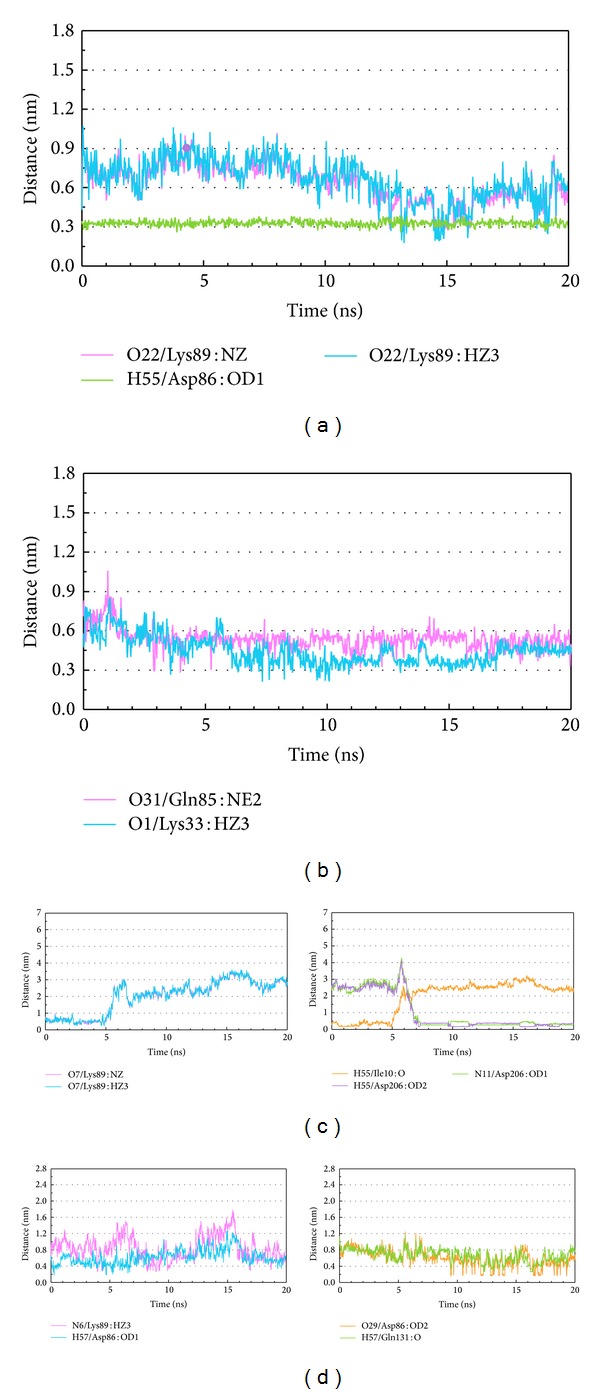
Distance of H-bond trajectories during MD simulation. (a) Tetrahydropalmatine, (b) Reserpiline, (c) (+)-Corydaline, and (d) Dinaciclib.

We illustrated distance of H-bond between the ligand and essential amino acids of CDK2 to discuss the binding force between the ligand and protein. According to occupancy of H-bond between the ligands and CDK2 protein, the ligands formed H-bonds with several residues of CDK2 protein permanently or temporarily. We picked up different patterns of distance of H-bond in the following description which did not necessarily appear in previous docking or MD poses. Tetrahydropalmatine formed H-bond with Lys89 of CDK2 unstably at late stage of MD but formed H-bond with Asp86 constantly at all stages of MD (Figure 11(a)). Reserpiline formed H-bond with Lys33 of CDK2 at middle stage of MD and formed H-bond with Gln85 unstably during MD (Figure 11(b)). (+)-Corydaline formed H-bond with Lys89 of CDK2 before 5 ns of MD. This compound also formed H-bond with Ile10 of CDK2 before 5 ns of MD but formed H-bond with Asp206 after 7 ns of MD instead (Figure 11(c)). The control formed H-bond with Asp86, Lys89, and Gln131 of CDK2 unstably during MD (Figure 11(d)). H-bond was important binding force between the ligand and protein. Based on these changes, we could discover that all the candidates and the control formed H-bonds with essential amino acids in quite different patterns.

**Figure 12:**
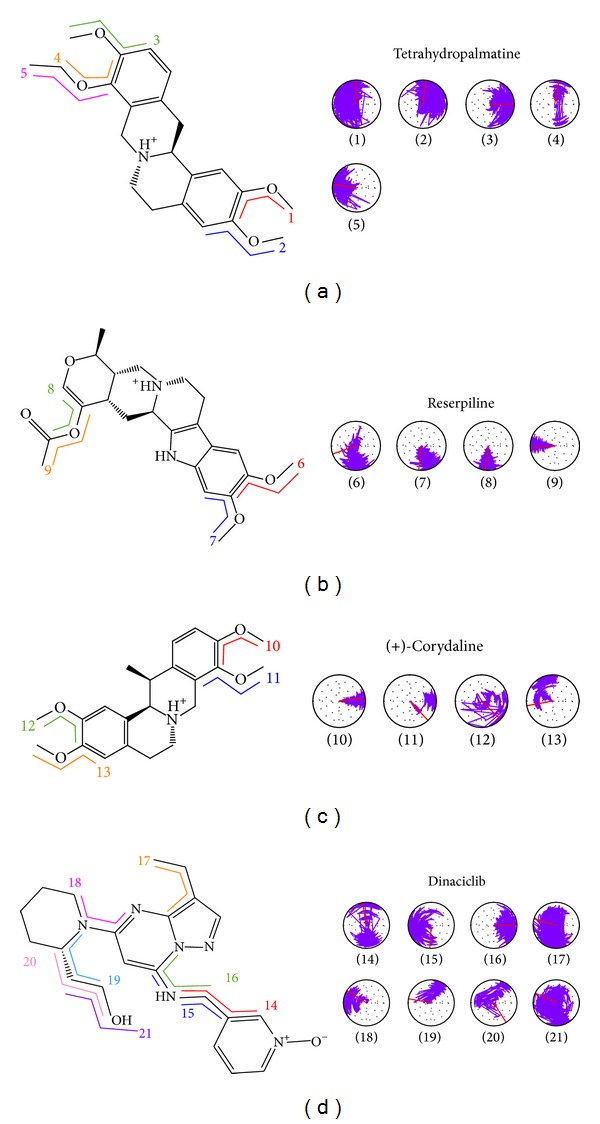
H-bond stability by torsion analysis. (a) Tetrahydropalmatine, (b) Reserpiline, (c) (+)-Corydaline, and (d) Dinaciclib.

The change in ligand torsion during MD also provided important clues to the stability of the H-bond. Tetrahydropalmatine formed 2 H-bonds with Lys89 of CDK2 as shown in Figures 3(a) and 4(a). The torsion angles 1 and 2 mean H-bonds of the ligand within 180-degree fluctuation (Figure 12(a)). Reserpiline formed H-bond with Lys89 as shown in Figures 3(b) and 4(b). The torsion angle 9 means stable H-bond of the ligand with tiny fluctuation (Figure 12(b)). (+)-Corydaline formed H-bond with Lys89 as shown in Figures 3(c) and 4(c). The torsion angle 13 means faint H-bond of the ligand. This finding was consistent with Figure 11(c). The temporary H-bond with Lys89 disappeared after 5 ns of MD (Figure 12(c)). The control formed H-bond with Ile10 as shown in Figures 3(d) and 4(d). The torsion angle 16 means H-bond within 90- degree fluctuation (Figure 12(d)).

**Figure 13:**
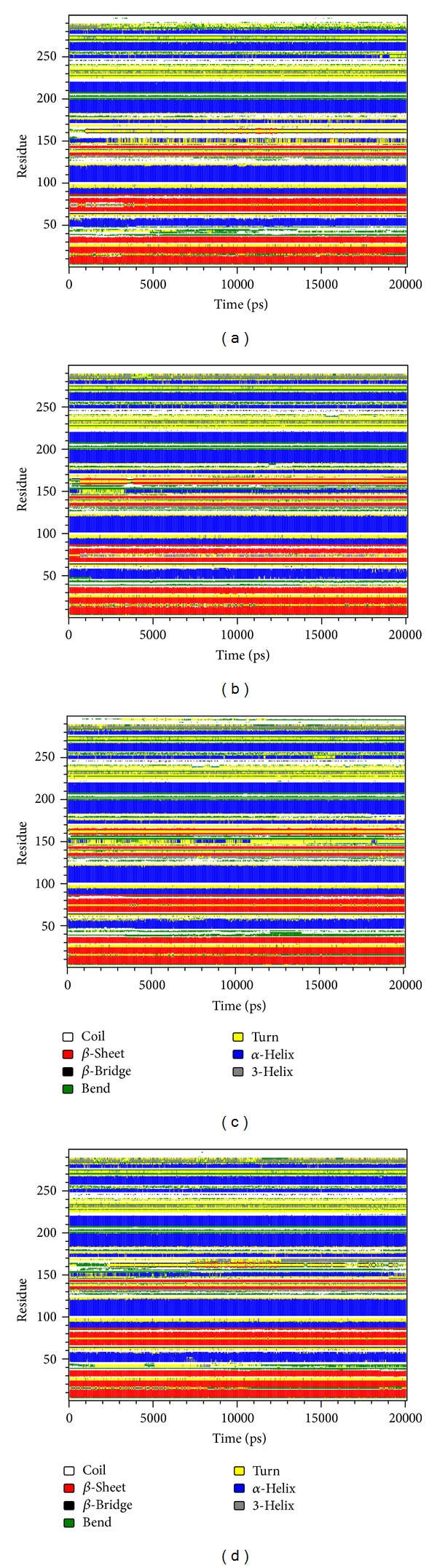
Secondary structure changes during MD simulation. (a) Tetrahydropalmatine, (b) Reserpiline, (c) (+)-Corydaline, and (d) Dinaciclib.

**Figure 14:**
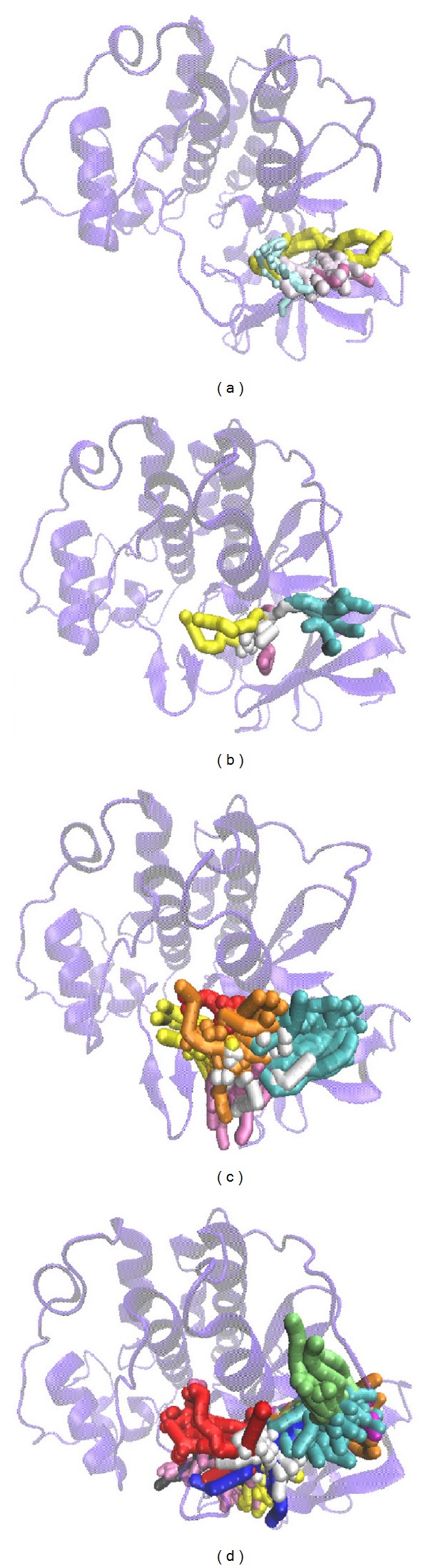
Analysis of transport pathways for CDK2 protein during MD simulation. (a) Tetrahydropalmatine, (b) Reserpiline, (c) (+)-Corydaline, and (d) Dinaciclib.

We drew secondary structure changes to discuss the change of structural component when the ligand bound with CDK2 protein. By observation of RMSF changes shown in Figure 8, the most fluctuated regions for the 3 candidates and the control were near residues 40 and 160. The findings of secondary structure changes were similar to that of RMSF. There were larger changes near residues 40 and 160, too (Figure 13). We speculated that all the 3 candidates and the control bound with CDK2 protein successfully and inhibited the activity of CDK2 by inducing its structural component change. We illustrated 3D simulation of ligand pathway to analyze all possible pathways when the ligand bound with CDK2 protein. There were 4 possible pathways for Tetrahydropalmatine or Reserpiline. However, there were 6 and 10 possible pathways for (+)-Corydaline and Dinaciclib, respectively (Figure 14).

## 4. Conclusion

One has found an important cell cycle controller. This guard can decide the cell cycle toward proliferation or quiescence. Normal cells follow the ordinary cycle, but cancer cells grow without rhythm. The rate of progress in cell cycle is regulated by cyclins and cyclin-dependent kinases (CDKs). CDK2 is a unique target among the CDK family in melanoma therapy. Previous studies had demonstrated that Dinaciclib was a CDK1/2/5/9 inhibitor leading to tumor apoptosis. We attempted to find out TCM compounds from the largest TCM Database@Taiwan in the world that have the ability to inhibit the activity of CDK2 by computational simulation.

We selected Tetrahydropalmatine, Reserpiline, and (+)-Corydaline as the candidates by docking and candidate screening results for further validation. Dinaciclib was assigned as the control compound. All the 3 candidates were better than the control in terms of docking score. Asp86 and Thr160 were the key residues for the 3 candidates and the control according to docking poses. All the 3 candidates were enrolled in constructing predicted activity using SVM, MLR models, and Bayesian network. These constructed models were reliable due to high *R* ^2^ values. The results suggested that predicted activity of any given compound was almost consistent with observed activity. Predicted activities of the 3 candidates were almost the same as or better than the control based on SVM, MLR, and BNT values.

MD simulation provided very useful information about the dynamic changes when the ligands bound with CDK2 protein. According to the figures of RMSD, Gyrate, MSD, and SASA, we concluded that the 3 candidates could bind with CDK2 and induce its conformational change the same as or even more stable than the control. Based on total energy and RMSF, we concluded that all the 3 candidates and the control bound with CDK2 protein stably but caused different fluctuation in individual residues. The MD poses of 0 ns and snapshot after 19 ns were compared with docking poses of ligand and protein. The results let us know the interesting change of binding sites during MD. We illustrated distance of H-bond and torsion analysis to observe how the important binding force affected the connection between the ligand and CDK2 protein. There were many interesting findings which had been described in the paper. The results of secondary structure changes were similar to that of RMSF. We speculated that all the 3 candidates and the control bound with CDK2 protein successfully and inhibited the activity of CDK2 by inducing its structural component change. Finally, 3D simulation of ligand pathway told us that there were many possible pathways when the ligand bound with CDK2 protein.

By overall analysis of docking results, predicted activity, and MD simulation, we could conclude that Tetrahydropalmatine, Reserpiline, and (+)-Corydaline had better binding affinity than Dinaciclib. All of them had the ability to inhibit the activity of CDK2 and might have the opportunity to be used in melanoma therapy.

## Figures and Tables

**Table 1:** Top 10 candidates of scoring function based on TCM Database@Taiwan screening.

Name	Dock score	H-bond key residues	H-bond quantity	Predicted activity		
				SVM∗	MLR∗	BNT∗
Tetrahydropalmatine	89.140	Asp86 and Lys89	3	6.233	6.156	6.509
Reserpiline	88.034	Asp86 and Lys89	2	7.148	6.044	6.995
(+)-Corydaline	86.231	Asp86 and Lys89	3	6.217	6.283	6.499
Taspine	85.957	Asp86	1	6.194	7.855	5.810
Thaliglucinone	83.869	Asp86	1	6.294	7.780	5.678
Hirsuteine	80.343	Asp86 and Lys89	2	6.308	5.494	6.519
Methoxymecambridine	79.702	Asp86 and Lys89	3	6.591	5.498	7.428
Evernic acid	78.944	Asp86 and Lys89	3	6.192	5.919	4.706
Strobilanthin	77.505	Glu8	2	5.686	5.095	3.338
Roxburghine X	76.897	Asp86 and Lys89	2	6.194	9.572	7.756
Dinaciclib∗	59.051	Ile10	1	6.405	3.229	6.899

Dinaciclib: control; SVM: support vector machine; MLR: multiple linear regression; BNT: Bayes Net Toolbox.
